# Regorafenib CSF Penetration, Efficacy, and MRI Patterns in Recurrent Malignant Glioma Patients

**DOI:** 10.3390/jcm8122031

**Published:** 2019-11-21

**Authors:** Pia S. Zeiner, Martina Kinzig, Iris Divé, Gabriele D. Maurer, Katharina Filipski, Patrick N. Harter, Christian Senft, Oliver Bähr, Elke Hattingen, Joachim P. Steinbach, Fritz Sörgel, Martin Voss, Eike Steidl, Michael W. Ronellenfitsch

**Affiliations:** 1 Dr. Senckenberg Institute of Neurooncology, University Hospital Frankfurt, Goethe University, 60528 Frankfurt am Main, Germany; pia.zeiner@kgu.de (P.S.Z.); Iris.Dive@kgu.de (I.D.); Gabriele.Maurer@kgu.de (G.D.M.); Oliver.Baehr@klinikum-ab-alz.de (O.B.); joachim.steinbach@med.uni-frankfurt.de (J.P.S.); Martin.Voss@kgu.de (M.V.); 2 University Cancer Center (UCT) Frankfurt, University Hospital Frankfurt, Goethe University, 60590 Frankfurt am Main, Germany; Katharina.Filipski@kgu.de (K.F.); patrick.harter@kgu.de (P.N.H.); Elke.Hattingen@kgu.de (E.H.); Eike.Steidl@kgu.de (E.S.); 3 German Cancer Consortium (DKTK), 60590 Frankfurt am Main, Germany; 4 Frankfurt Cancer Institute (FCI), University Hospital Frankfurt, Goethe University, 60590 Frankfurt am Main, Germany; 5 IBMP—Institute for Biomedical and Pharmaceutical Research, 90562 Nürnberg-Heroldsberg, Germany; Martina.Kinzig@ibmp.net (M.K.); Fritz.Soergel@ibmp.net (F.S.); 6 Institute of Neurology (Edinger-Institute), University Hospital Frankfurt, Goethe University, 60528 Frankfurt am Main, Germany; 7 Department of Neurosurgery, University Hospital Frankfurt, Goethe University, 60528 Frankfurt am Main, Germany; christian.senft@kgu.de; 8 Department of Neurology, Klinikum Aschaffenburg-Alzenau, 63739 Aschaffenburg, Germany; 9 Department of Neuroradiology, University Hospital Frankfurt, Goethe University, 60528 Frankfurt am Main, Germany; 10 Institute of Pharmacology, University Duisburg-Essen, 45141 Essen, Germany

**Keywords:** malignant glioma, glioblastoma, regorafenib, regorafenib csf concentration, MRI patterns of gliomas

## Abstract

(1) Background: The phase 2 Regorafenib in Relapsed Glioblastoma (REGOMA) trial indicated a survival benefit for patients with first recurrence of a glioblastoma when treated with the multikinase inhibitor regorafenib (REG) instead of lomustine. The aim of this retrospective study was to investigate REG penetration to cerebrospinal fluid (CSF), treatment efficacy, and effects on magnetic resonance imaging (MRI) in patients with recurrent high-grade gliomas. (2) Methods: Patients were characterized by histology, adverse events, steroid treatment, overall survival (OS), and MRI growth pattern. REG and its two active metabolites were quantified by liquid chromatography/tandem mass spectrometry in patients’ serum and CSF. (3) Results: 21 patients mainly with IDH-wildtype glioblastomas who had been treated with REG were retrospectively identified. Thirteen CFS samples collected from 3 patients of the cohort were available for pharmacokinetic testing. CSF levels of REG and its metabolites were significantly lower than in serum. Follow-up MRI was available in 19 patients and showed progressive disease (PD) in all but 2 patients. Two distinct MRI patterns were identified: 7 patients showed classic PD with progression of contrast enhancing lesions, whereas 11 patients showed a T2-dominant MRI pattern characterized by a marked reduction of contrast enhancement. Median OS was significantly better in patients with a T2-dominant growth pattern (10 vs. 27 weeks respectively, *p* = 0.003). Diffusion restrictions were observed in 13 patients. (4) Conclusion: REG and its metabolites were detectable in CSF. A distinct MRI pattern that might be associated with an improved OS was observed in half of the patient cohort. Treatment response in the total cohort was poor.

## 1. Introduction

Malignant glioma is a highly lethal disease despite multimodal treatment approaches with a median overall survival (OS) of less than 15 months in glioblastoma patients under standard treatment comprising radiotherapy plus concomitant and adjuvant temozolomide chemotherapy [1]. For recurrent glioblastoma, there is yet no standard therapy [2]. Targeted therapies as well as antiangiogenic agents like bevacizumab (BEV) have failed to improve OS [3,4,5]. Nevertheless, antiangiogenic treatments are biologically active and BEV can induce distinct magnetic resonance imaging (MRI) patterns including a decrease in contrast enhancement and T2 hyperintense edema [6]. These alterations required a revision of the response assessment in neuro-oncology (RANO) criteria to include non-enhancing T2/fluid attenuated inversion recovery (FLAIR) lesions as a new criterion for glioma progression [7]. Regorafenib (REG) is a multikinase inhibitor that inhibits, amongst others, the vascular endothelial growth factor (VEGF) receptors 1–3 [8] and has shown efficacy in several cancers [9,10,11] as well as preclinical glioma models [12,13]. In the phase 2 Regorafenib in Relapsed Glioblastoma (REGOMA) trial for first recurrence of a glioblastoma, REG increased the median OS from 5.6 to 7.4 months compared to lomustine [14]. However, these results have been challenged because of the unusually short OS in the lomustine arm [15]. Two small retrospective analyses in late-stage high-grade glioma patients reported poor REG treatment response and significant adverse events (AEs) [16,17]. In a cohort of six glioma patients no impact of REG on MRI findings was observed [16].

We report the results of a retrospective analysis of 21 high-grade recurrent glioma patients treated with REG. We demonstrate that REG and its active metabolites can be detected in the CSF. Additionally, we describe distinct MRI alterations that might correlate with a survival benefit in patients undergoing REG treatment.

## 2. Materials and Methods

### 2.1. Study Design and Population

We performed a systematic retrospective observational study of all patients with recurrent high-grade gliomas that were treated with REG between August 2018 and July 2019 at the Dr. Senckenberg Institute of Neurooncology, University Hospital Frankfurt, Goethe University, Germany. Demographic and clinical data were extracted from patients’ records, deidentified, and entered into password-protected databases. Tumors were diagnosed at the Neuropathology Department (Edinger Institute) by two experienced neuropathologists (K.F., P.N.H.). Tumors of 10 patients were additionally classified via DNA methylation-based profiling (Infinium 850 k EPIC array) [18]. Otherwise, the assessment of 1p/19q co-deletion was performed by chromogenic in situ hybridization (CISH), O6-methylguanine-DNA methyltransferase (MGMT) gene promoter methylation was evaluated by a methylation-specific PCR protocol and the IDH1R132H-mutation status was investigated via immunohistochemical analysis.

Glioma recurrence prior to initiation of REG treatment was confirmed by two experienced neuroradiologists (E.S., E.H.) based on MRI scans at the Department of Neuroradiology. The use of patient material and data was approved by the ethics committee of the University Hospital Frankfurt (SNO-3-2019).

### 2.2. Follow-Up and Statistical Analysis

Data were collected retrospectively from follow-up appointments documented in patients’ records in an observational period between August 2018 and July 2019. Missing or incomplete follow-up data are indicated. Descriptive statistics (median, range, and percentages) were used to summarize demographics, AEs, as well as steroid reduction under REG treatment. OS, defined as the time from initiation of REG treatment until death from any cause, was determined by Kaplan-Meier analysis and the log-rank test (GraphPad Prism 8).

### 2.3. Neuroimaging 

MRI scans were acquired before (baseline) and on average in 6-week intervals after the initiation of REG treatment (follow-up, range 12–77 days for first follow up) on both 1.5 and 3 Tesla MRI scanners. The minimum scope of sequences of all examinations was based on current recommendations for monitoring of brain tumor patients [19]. Based on the RANO criteria [7], treatment response was assessed comparing the follow-up to the baseline MRI by two experienced neuroradiologists (E.S., E.H.). Following criteria suggested in the original RANO publication [7] and employed in other studies [20], we visually discriminated non-enhancing tumor from peritumoral edema on T2 and FLAIR sequences. Changes in edema size were additionally evaluated visually. Furthermore, we analyzed apparent diffusion coefficient (ADC) maps derived from diffusion weighted imaging (DWI) with at least two b values. The minimum ADC values in the tumor were measured and normalized by the ADC values in the contralateral, normal appearing tissue (ADCratio). This standard procedure has been previously described [21,22]. For examinations including a dynamic susceptibility contrast (DSC) MR perfusion measurement (single bolus of Gd-DO3A-butrol (Gadovist^®^, Bayer Vital GmbH Germany), 0.1 mmol/kg bodyweight), we analyzed the maximum cerebral blood volume in the tumor in relation to the cerebral blood volume in the normal appearing, contralateral tissue (relative maximum cerebral blood volume, rCBVmax) as a standard parameter [23]. The analysis was performed using the MR Neuro Perfusion application within the Philips IntelliSpace^®^ software toolbox, which employs the Boxerman-Weisskoff approach to correct for contrast agent leakage [24]. Patients were categorized into a (1) classic progressive disease (PD) pattern (defined by a 25% or more increase in enhancing lesions on T1 post-contrast imaging) or (2) a T2-dominant growth pattern with an overall decrease in contrast enhancement on T1 post-contrast imaging (though new appearing, dot-like enhancement was possible) and a distinct increase in non-enhancing tumor on T2 imaging (Figure S1). Additionally, MRI precontrast T1 sequences and available computed tomography (CT) scans were assessed for new intratumoral calcifications.

### 2.4. Collection of Serum and Cerebrospinal Fluid Samples of Patients

CSF was obtained by puncture of a Rickham reservoir (patient 5; 11 samples, 3 prior to initiation of REG therapy) or lumbar puncture (patient 17 and 18; 1 sample each after initiation of REG therapy), immediately centrifuged at 300 g for 10 min (Eppendorf centrifuge 5702R) to remove cellular components and transferred into liquid nitrogen for storage. Additional CSF parameters including cell count, lactate, glucose, protein, and albumin were recorded. If available, remaining patient serum was also stored in liquid nitrogen for later analysis.

### 2.5. Liquid Chromatography/Tandem Mass Spectrometry-Based Quantification of Regorafenib and its Active Metabolites

A detailed description of the performed liquid chromatography/tandem mass spectrometry (LC-MS/MS) method for REG quantification is included in Appendix A.

## 3. Results

### 3.1. Patient Characteristics and Treatment

A total of 21 patients undergoing REG treatment for recurrent malignant gliomas were identified (Table 1). Mutation status of isocitrate dehydrogenase (IDH) and methylation status of the MGMT gene promoter were available in 19 patients of our cohort (Table 1). One tumor displayed 1p/19q co-deletion indicating oligodendroglioma. The patient cohort comprised heavily pre-treated patients—all patients had received prior alkylating chemotherapy (temozolomide or temozolomide in combination with lomustine) and radiation in the first line setting and had suffered a median of 2 previous tumor recurrences (Table 1). Only 4 patients suffered from a first recurrence in accordance to the inclusion criteria of the REGOMA trial [14]. All patients received REG as a last line treatment. Antiangiogenic therapy with BEV prior to REG had been administered in 3 patients (patient 6, 12 and 16, with only patient 16 having a follow-up MRI for analysis). The treatment schedule was identical to the REGOMA trial with a REG dose of 160 mg per day for 21 consecutive days followed by a pause of 7 days [14]. In case of unacceptable toxicity or clinical deterioration, a dose reduction to 120 mg was performed. Median time since initial tumor diagnosis to initiation of REG was 553 days (217–4525 days) indicating the late disease stage in our patient cohort (Table 1). None of the patients received salvage chemotherapy after failure of REG.

### 3.2. Detection of Regorafenib and its Metabolites in Cerebrospinal Fluid

In humans, REG is a CYP3A4 substrate and primarily metabolized in the liver to form two metabolites, a N-oxide derivative and a demethylated N-oxide derivative [25] with similar kinase inhibitory properties [26]. To our knowledge, no data on the CSF distribution of REG and its metabolites have been published so far.

**Figure 1:**
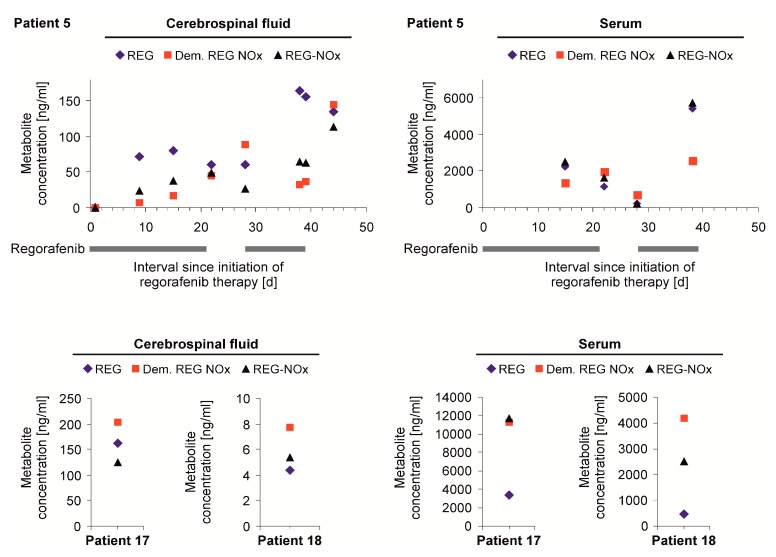
Cerebrospinal fluid (CSF) and serum levels of regorafenib (REG). CSF and serum of patients 5, 17, and 18 was preserved during several time points of REG treatment. The concentrations of REG (blue diamonds) and the active REG metabolites demethylated REG N-oxide (red squares) and REG N-oxide (black triangles) are depicted. Ventricular CSF of patient 5 was obtained via Rickham reservoir puncture at different time points during treatment with REG (indicated by black bars, upper panel). For patients 17 and 18, only one sample at a single time point was available (lower panel). Lumbar puncture was performed in patient 17 on the first day of the second cycle of REG treatment (4.5 h after administration). In patient 18, the lumbar puncture was performed on day 22 of the first cycle, however the patient stopped REG after day 19 (>48 h after the last administration).

We collected CSF and serum samples from 3 patients (patients 5, 17, and 18) and REG and its active metabolites were detectable in both serum and CSF. Levels of REG and its metabolites were approximately by a factor between 4 and 540 higher in serum than in CSF. Because patient 5 required repeated punctures of the Rickham reservoir due to occlusive hydrocephalus (Figure S2), CFS levels could be monitored over time (Figure 1). CSF was collected before (*n* = 3) and after initiation of REG treatment (*n* = 8). REG and its metabolites were not detectable in the first CSF sample that was obtained only 3 h after the first administration of REG (no serum sample was available for this time point). There was a trend towards higher metabolite concentrations in the second REG cycle (Figure 1) that had to be terminated prematurely due to clinical deterioration. CSF from patients 17 and 18 was obtained by lumbar puncture after initiation of REG. While REG itself was the dominating metabolite in the CSF of patient 5 in 5 of 7 samples, demethylated N-oxide was the most prevalent metabolite in the other 2 patients (Figure 1). Lumbar puncture of patient 18 was performed 2 days after the last REG intake which most likely is the cause for the comparatively low concentrations. In all available serum samples, the REG metabolites were usually more prevalent than REG itself.

### 3.3. MRI Growth Patterns under Regorafenib Treatment

At least one follow-up MRI was acquired for 19 patients. Detailed MRI characteristics were recorded (Table S1). Based on RANO criteria, 17 patients had PD and 2 patients had stable disease (SD) upon follow-up MRI. In 10 out of 12 patients with available DSC perfusion measurements, the rCBVmax was increased at least 2.5-fold (Table S1). 2 or more consecutive follow-ups were obtained in 6 patients. 4 of 6 patients with a second follow-up showed continued tumor growth, in accordance with the initial follow-up MRI. Patient 16 had previously also been rated SD. In contrast, patient 8 had previously shown tumor growth, but since the first follow-up was only 23 days into REGO treatment, the patient was overall rated SD according to RANO (Figure S3).

**Figure 2:**
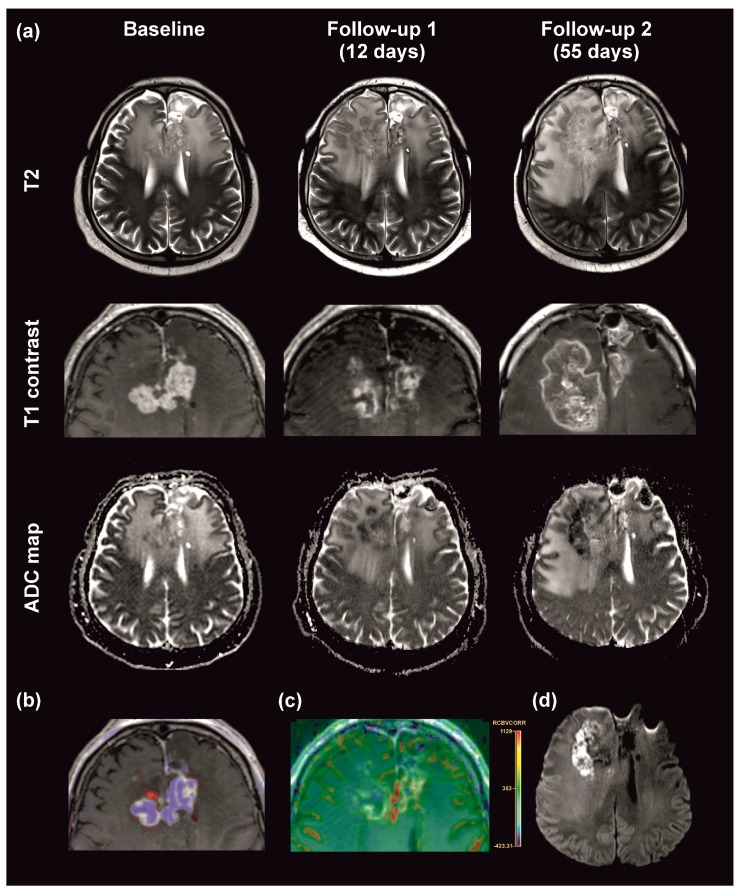
Magnetic resonance imaging (MRI) alterations in a regorafenib-induced T2-dominant progression pattern. (**a**) MRI scans were performed at baseline and the indicated intervals after initiation of REG therapy. T2 weighted images (T2, upper row), contrast enhanced T1 weighted images (T1 contrast, middle row), and apparent diffusion coefficients (ADC) maps (lower row) are depicted. “T2-dominant” progression is characterized by an overall decrease of contrast-enhancement and simultaneous increase in T2 hypointense tumor areas in the right frontal lobe. (**b**) Comparison of T1 contrast images at baseline and after 12 days. Areas of decreasing signal intensity are segmented in blue, areas of increasing intensity are segmented in red. (**c**) Color-coded perfusion map for leakage corrected cerebral blood volume (CBV) showing patchy, red areas with markedly increased CBV within the tumor outside of areas with reduced ADC. (**d**) Diffusion-weighted b 1000 image corresponding to corresponding ADC map at 55 days.

Two different MRI growth patterns under REG treatment were identified: 7 patients presented with classic PD and 11 patients with a T2-dominant growth pattern. Patient 16, who had been pretreated with BEV, showed no changes in follow-up MRIs and was therefore not considered for subgroup analyses. The follow-up MRI of patient 1 classified as a T2-dominant growth pattern exemplarily illustrates the low T2 signal intensity of cell dense tumor tissue infiltrating the adjacent cortex that is well distinguishable from the hyperintense, perifocal edema and exceeds the area of contrast enhancement (Figure 2a, upper row). Even though patients with a T2-dominant pattern showed an overall reduction in contrast enhancement, the outer margin of lesions often kept discreetly growing (Figure 2a, middle row). MRI alterations under REG treatment were detectable as early as 12 days after the initiation of REG (Figure 2a).

### 3.4. Cerebral Diffusion Weighted Imaging Lesions in Regorafenib-Treated Patients

We have previously reported DWI lesions in patients receiving antiangiogenic therapy with the VEGF-A antibody BEV [21,27]. Because REG targets VEGF receptors, we hypothesized that REG might cause similar DWI lesions. Indeed, 13 patients developed new diffusion restrictions with a low ADC ratio within the tumor (Figure 2a lower row, Figure 2b–d, Figure S4, Table S1). ADC of patient 2 could not be analyzed due to artifacts. Notably, patient 15 had already shown small, intratumoral diffusion restrictions at baseline. Diffusion restricted lesions generally had a patchy pattern and no rCBV increase. Extensive diffusion restrictions were only seen in patient 20. Pre-contrast T1 sequences and CT scans (available in 10 patients) showed corresponding, new hyperintensities/hyperdensities only in patient 21.

### 3.5. Efficacy and Safety Profile of Regorafenib in Recurrent Malignant Glioma Patients

All but two patients, who both terminated REG after 20 instead of the planned 21 days, completed at least one cycle of REG therapy. Median duration of REG therapy was 45 days (range 19–139) (Table 2). Dose reduction from 160 to 120 mg daily was necessary in 4 patients due to adverse events (AEs) including diarrhea (patient 7), hand-foot syndrome and sub-febrile temperatures (patient 12), mucositis and fatigue (patient 15), and mucositic glossodynia and leukopenia (patient 16) (Table 2). The most frequent AEs were of grade 1 according to the Common Terminology Criteria for Adverse Events (CTCAE) and included skin reactions (hand-foot syndrome, rash, oral ulcers), elevated transaminases, fatigue, infections, and myelosuppression. Patients 7 and 12 suffered lung-artery embolization as the most serious AEs (CTCAE grade 3) observed under treatment with REG in our cohort (Table 2).

**Figure 3:**
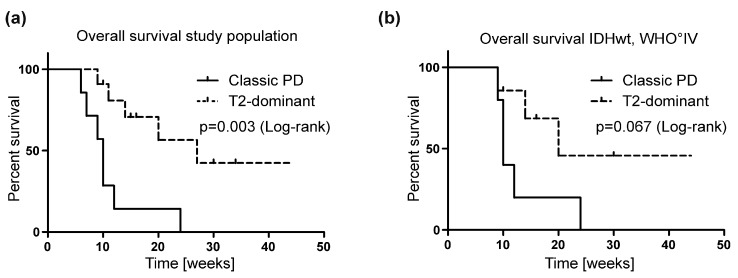
Association of magnetic resonance imaging (MRI) progression patterns with overall survival. Kaplan-Meier survival curves were obtained by discriminating classic progressive disease (PD) in contrast to T2-dominant growth patterns (T2-dominant) in MRI as indicated in the figure. Curves were compared by log-rank (Mantel-Cox) test (*p*-values depicted). (**a**) Overall survival for patients with classic PD (*n* = 7) and T2-dominant growth pattern (*n* = 11) (median 10 vs. 27 weeks). (**b**) Overall survival for the subcohort of patients with IDH-wildtype (wt) WHO IV glioblastoma (classic PD *n* = 5, T2-dominant growth pattern *n* = 7) (median 10 vs. 20 weeks).

The occurrence of the abovementioned T2-dominant MRI growth pattern was associated with a significantly better median OS in contrast to patients with a classic PD pattern. Median OS from initiation of REG treatment was 14 weeks in the total cohort of 21 patients. In the subgroup of 11 patients with a T2-dominant growth pattern, OS was 27 weeks in contrast to the OS of 10 weeks in 7 patients with classic PD (*p* = 0.003 **) (Figure 3a, Table 3). However, when considering only glioblastomas of our cohort, this effect was only detectable as a trend (*p* = 0.067) (Figure 3b), potentially due to the small group size.

An overall decrease of the peritumoral edema without an increase of steroid dosage was observed in 6 patients. In several patients with multilocular tumors, simultaneous increases and decreases of edema around different lesions were detectable (Figure 2a, upper row, continuous decrease in the left frontal lobe and increase in the contralateral side). There was no strict correlation between the reduction of contrast enhancement and edema (Table S1).

## 4. Discussion

Treatment of recurrent gliomas remains a major challenge of daily neuro-oncology practice. Frequently, patients in good clinical condition are confronted with an exhausted therapeutic arsenal. Therefore, new treatment approaches are urgently needed. The phase 2 REGOMA trial investigating the multikinase inhibitor REG in recurrent glioblastoma sparked hope for the successful employment of a targeted therapy approach in gliomas [14]. A major point of criticism, besides the unexpectedly bad course of disease in the lomustine control group (median OS of 5.6 months), was the lack of data for blood-brain barrier penetration of REG and its active metabolites [15]. REG does not only target VEGF receptors localized at the endothelium, but might additionally interact with molecules at the tumor cell surface [8], which emphasizes the question if the drug penetrates the blood-brain barrier.

In our retrospective study, we demonstrate for the first time that REG and its metabolites are detectable in low levels in patients’ CSF. We provide the respective concentrations in a total of 10 CSF samples of three different patients (Figure 1). While REG itself was the main component in the ventricular CSF of patient 5 (Figure 1, upper panel), the levels of the two metabolites were higher in the patients investigated by lumbar puncture (Figure 1, lower panel). Previous pharmacokinetic studies indicated that REG is the main component and that REG N-oxide is the major REG metabolite in plasma [28], whereas in our serum measurements the metabolite REG N-oxide surpassed the concentrations of REG itself. In vitro data suggest an efficacy of REG and its metabolites in the nanomolar (nM) range depending on the target kinase and model [8]. The inhibitory concentrations 50 (IC50) in a cell culture assay were rather low at 3 + 2 nM for the VEGF receptor 2, but higher for other REG targets like Tie2 with concentrations of 31 + 9 nM [8]. In our analyses, REG CSF concentrations were significantly lower than serum concentrations and ranged between 10 and 100 ng/ml corresponding to 21 and 207 nM, respectively (Figure 1). Thus, it seems plausible that a sufficient CNS concentration of REG and its metabolites could be reached in glioma patients at least temporarily. However, during the course of a regular REG cycle with 21 days of dosing followed by 7 days of pause, the REG concentration could drop to levels below the nM range as evidenced in patient 18 with a REG level of 4 ng/ml (8 nM) 2 days after finishing the dosing for the first cycle. The main limitation of this analysis is that drug concentrations assessed in the CSF can only approximate the concentration of REG in the tumor tissue itself and might rather reflect concentrations at the edge of the parenchymal brain tissue [29]. Studies including more accurate techniques like intracerebral microdialysis monitoring could provide a more detailed understanding [30].

Based on RANO criteria, all but 2 of 19 patients available for follow-up MRI were classified as PD upon follow-up. This outcome is worse than reported by both the REGOMA trial and Tzaridis et al. [14,17] and could be due to the less favorable patient characteristics and short OS in our cohort. Nevertheless, distinct MRI alterations were observed in half of the patients. A reduction of contrast enhancement despite a progression of non-enhancing tumor lesions was noted in 10 patients. This T2-dominant growth pattern (Table S1, Figure S1) partially resembled MRI patterns described for BEV treatment [21]. Due to the retrospective nature of our analysis, we cannot rule out that the differences in tumor grade and molecular tumor characteristics contribute to this type of progression pattern. A change from a local to a diffuse growth type was not associated with worse outcome under BEV treatment [31] and a diffuse T2 progression pattern was even identified to be associated with improved OS in another cohort [32]. In our total cohort of 21 patients with late-stage malignant gliomas that had undergone a median of 2 (1–5) prior treatment regimens, no distinct indication of clinical efficacy was observed in contrast to the patients of the REGOMA trial (median OS of 14 weeks). However, those patients with a T2-dominant growth pattern showed a significantly better median OS of 27 weeks from initiation of REG treatment in contrast to patients with classic PD that had a median OS of 10 weeks (*p* = 0.003) (Figure 3). This effect was still detectable when excluding the patient with the 1p19q co-deleted oligodendroglioma. Notably, IDH-mutant glioma patients were similarly distributed in the two subgroups (Table 1). None of the noted effects were described by the only other study analyzing MR imaging during REG treatment by Kebir et al. [16]. This could be explained by the small sample size of 6 patients in the study by Kebir et al. with half of the patients receiving additional tumor therapies including surgery and radiotherapy. Also, the follow-up interval in the study by Kebir et al. was longer, reaching up to 4 months. Yet it has to be noted that for most patients that received more than one follow-up MRI in this study, the described effects were still visible in the second MRI. Our results also indicate the necessity for predictive biomarkers for treatment response as well as for a potential correlation with neuroradiological (response) patterns [33]. Biomarker identification platforms including proteomic approaches offer novel opportunities [34] and ideally biosignatures for monitoring the response to neurooncological treatments could be implemented in future clinical trials [33,34]. In this regard, effects of early responses to REG treatment in an animal model measured by dynamic contrast-enhanced computed tomography have been previously described [35].

Considering the survival difference in our relatively small cohort, a potential prognostic role of the MRI patterns under REG or other VEGF-targeting therapies should be confirmed at an earlier stage of disease and in larger cohorts of prospective future studies. Clearly, the retrospective approach, the size, and the heterogeneity of our heavily pre-treated patient cohort were limitations in terms of the analysis of causal relations.

Another controversially discussed imaging feature during antiangiogenic therapy with the VEGF-A antibody BEV is the appearance of “stroke-like” DWI restrictions within glioblastomas. Several studies have found these lesions to be associated with either prolonged or reduced OS [36,37,38]. With corresponding T1-hyperintensities or calcifications detected in CT scans, these lesions were identified as favorable biomarkers under BEV treatment by a previous study of our institute [39]. Because REG targets VEGF receptors, we hypothesized that this treatment might cause similar alterations. Indeed, 13 patients developed marked diffusion restrictions, but only one patient showed additional T1-hyperintensities/calcifications. As another likely antiangiogenic imaging feature, we noticed a reduction of the peritumoral edema in 6 patients. Within an individual tumor, perifocal edema of different lesions could develop divergently, limiting the overall effect. Overall, the potency of REG for edema reduction seems limited as compared to BEV, which induced edema reduction in 88% of patients [40]. In accordance, we only observed a steroid reduction under REG treatment in 2 of 21 patients.

## 5. Conclusions

REG and its active metabolites reached low CSF levels and some biological activity was observed resulting in distinct MRI growth patterns that might be mediated either by REG effects on endothelial or tumor cells in the brain parenchyma. A T2-dominant growth pattern occurred in half of our patients and might be associated with a survival benefit. However, overall treatment response to REG was poor in our cohort of patients with late-stage malignant gliomas. Especially in the light of the observed AEs, our results mandate caution when considering REG as a last line salvage treatment. Additional clinical trials are needed to further investigate REG efficacy and the prognostic relevance of REG-induced MRI alterations.

## Figures and Tables

**Table 1:** Patient characteristics, previous treatments, concomitant therapy, and magnetic resonance imaging (MRI) patterns.

Characteristics	All Patients (*n* = 21)	Classic (*n* = 7, 33%)	T2-Dominant (*n* = 11, 52%)
**General**			
Age, y, median (range)	49 (37–80)	40 (37–80)	53 (38–72)
Female, % (*n*)	38 (8)	29 (2)	45 (5)
Karnofsky performance status scale, %, median (range)	70 (50–100)	80 (50–90)	70 (50–100)
**Histology, % (*n*)**			
Isocitrate dehydrogenase (IDH)-wildtype	71 (15)	71 (5)	73 (8)
IDH-mutant	19 (4)	29 (2)	18 (2)
IDH unknown	10 (2)	0 (0)	9 (1)
1p/19q co-deletion	5 (1)	0 (0)	9 (1)
WHO IV	81 (17)	86 (6)	73 (8)
WHO III	19 (4)	14 (1)	24 (3)
**O6-methylguanin-DNA methyltransferase (MGMT) status, % (*n*)**			
Unmethylated	33 (7)	43 (3)	27 (3)
Methylated	38 (8)	29 (2)	55 (6)
Inconclusive	19 (4)	14 (1)	9 (1)
**Surgery**			
No. of resections, median (range)	1 (0–3)	1 (1–2)	1 (0–2)
Patients with 1 resection, % (*n*)	62 (13)	86 (6)	55 (6)
Patients with biopsy only, % (*n*)	5 (1)	0 (0)	9 (1)
**Previous Radiotherapy**			
No. of radiotherapies, median (range)	2 (1–3)	2 (1–2)	1 (1–2)
Patients without radiotherapy, % (*n*)	0 (0)	0 (0)	0 (0)
**Previous chemotherapy**			
No. of chemotherapies, median (range)	2 (1–5)	2 (1–3)	2 (1–5)
Previous NovoTTF-100A, % (*n*)	24 (5)	14 (1)	27 (3)
**Recurrences**			
No. of recurrences, median (range)	2 (1–7)	2 (1–4)	2 (1–6)
Patients with 1 recurrence, % (*n*)	19 (4)	14 (1)	27 (3)
**Time from diagnosis to REG, days, median (range)**	553 (217–4525)	561 (269–4515)	508 (217–4384)
**Concomitant therapy, % (*n*)**			
Regorafenib (REG) monotherapy	90 (19)	86 (6)	91 (10)
REG + radiotherapy	5 (1)	0 (0)	9 (1)
REG + temozolomide	5 (1)	14 (1)	0 (0)
**Time to first MRI after initiation of REG, days, median (range)**	49 (12–77)	44 (20–56)	49 (12–77)

**Table 2:** Safety profile.

Characteristics	All Patients (*n* = 21)	Classic (*n* = 7, 33%)	T2-Dominant (*n* = 11, 52%)
Treatment, days, median (range)	45 (19–139)	37 (19–55)	56 (28–139)
Regorafenib dose reduction, % (*n*)	19 (4)	0 (0)	18 (2)
Adverse Events, % (*n*)			
Skin reaction	33 (7)	29 (2)	27 (3)
Elevated transaminases	29 (6)	29 (2)	36 (4)
Fatigue	19 (4)	14 (1)	18 (2)
Infection	19 (4)	14 (1)	9 (1)
Myelosuppression	19 (4)	29 (2)	9 (1)
Lung-artery embolization	10 (2)	0 (0)	9 (1)

**Table 3:** Regorafenib efficacy.

Characteristics	All Patients (*n* = 21)	Classic (*n* = 7, 33%)	T2-Dominant (*n* = 11, 52%)
Steroid reduction, % (*n*)	10 (2)	0 (0)	18 (2)
Overall survival, weeks, median (range)			
Total cohort	14 (4–44)	10 (6–24)	27 (9–44)
Censored patients, % *(n* of X)	33 (7 of 21)	0 (0 of 7)	55 (6 of 11)
IDH-wildtype WHO IV patients		10 (9–24)	20 (9–44)
Censored patients, % (*n* of X)		0 (0 of 5)	57 (4 of 7)

## Supplementary Materials

The following are available online at https://www.mdpi.com/2077-0383/8/12/2031/s1, Table S1: Patient and imaging characteristics for first MRI follow-up during REG treatment, Figure S1: Patterns of malignant glioma progression, Figure S2: Rickham reservoir implantation in a patient with occlusion hydrocephalus due to a glioblastoma, Figure S3: MR imaging dynamics of patient 8, Figure S4: Stroke-like lesions in regorafenib-treated patients.

## Appendix A

### LC-MS/MS-Based Quantification of Regorafenib and its Active Metabolites

Regorafinib, N-desmethyl-regorafenib-(pyridine)-N-oxide, and Regorafenib (Pyridine)-N-oxide concentrations were quantified in human serum and CSF by a liquid chromatography/tandem mass spectrometry (LC-MS/MS) method. The liquid chromatography systems consisted of a binary LC-pump (Agilent 1260 Infinity, Agilent Technologies, Waldbronn, Germany) and an analytical column (Phenomenex Kinetex 2.5 µm F5, 50 × 4.6 mm, Phenomenex LTD, Aschaffenburg, Germany). Gradient elution was performed with 0.1% formic acid and 0.1% formic acid in acetonitrile. Determination was performed using a SCIEX Triple Quad™ 6500+ mass spectrometer (SCIEX, Concord, Ontario, Canada) and Analyst^®^ software version 1.7 (SCIEX, Concord, Ontario, Canada). Fifty µL of each human serum sample was placed in a polypropylene-tube. Samples were deproteinized with 300 µL acetonitrile (containing the internal standard moxifloxacin-d5), subsequently vortex-shaked and centrifuged. The supernatant was further diluted with Milli-Q^®^ water and 10 µL of each sample was injected into the LC-MS/MS system. Fifty µL of each human CSF sample was placed in a polypropylene-tube. Samples were deproteinized with 200 µL (regorafinib) or 300 µL (N-desmethyl-regorafenib-(pyridine)-N-oxide and regorafinib-(pyridine)-N-oxide) acetonitrile (containing the internal standard moxifloxacin-d5), subsequently vortex-shaked and centrifuged. The supernatant was further diluted with Milli-Q^®^ water and 10 µL of each sample was injected into the LC-MS/MS system. The samples for regorafenib, N-desmethyl-regorafenib-(pyridine)-N-oxide, and regorafenib-(pyridine)-N-oxide were detected with MRM (multiple reaction monitoring) as follows: precursor → product ion for regorafinib m/z 483.1 → 270.1, N-desmethyl-regorafenib-(pyridine)-N-oxide m/z 485.8 → 425.0, regorafenib-(pyridine)-N-oxide m/z 499.8 → 305.2, and for moxifloxacin-d5 (internal standard) 407.4 → 266.4 m/z; for all analytes in positive mode. Under these conditions regorafenib, N-desmethyl-regorafenib-(pyridine)-N-oxide, regorafenib-(pyridine)-N-oxide, and the internal standard eluted after approximately 2.99, 2.75, 2.83 and 1.44 min.

Calibration standards and spiked quality controls samples (SQC) were prepared by adding a defined amount of analyte-solutions to human drug-free serum or CSF. Calibration was performed by weighted (1/concentration2) linear regression. Linearity for regorafinib, N-desmethyl-regorafenib-(pyridine)-N-oxide, regorafenib-(pyridine)-N-oxide could be demonstrated over a calibration range from 5.590 to 5031 ng/mL, 1.343 to 604.3 ng/mL, and 1.328 to 597.5 ng/mL in serum and from 0.9287 to 105.6 ng/mL, 1.070 to 102.7 ng/mL, and 1.058 to 101.6 ng/mL in CSF. No interferences were observed for the three analytes and the internal standard in both matrices.

The precision and accuracy for the regorafenib SQCs in human serum ranged from 3.8% to 7.9% and 92.1% to 101.1 %. In human CSF, precision and accuracy ranged from 4.1% to 5.6% and 99.5% to 101.4%. The mean assay variance of the six human serum samples evaluated for incurred samples reanalysis for regorafinib was 4.1%. The mean assay variance of the all thirteen human CSF samples evaluated for incurred samples reanalysis for regorafenib was 5.2%. No sample value beyond ±20% deviation from the mean was observed in human serum and CSF. Incurred sample reanalysis in human serum and CSF showed reliability of the assay for regorafinib.

The precision and accuracy for the N-desmethyl-regorafenib-(pyridine)-N-oxide SQCs in human serum ranged from 2.5% to 9.4% and 92.2% to 101.9%. In human CSF, precision and accuracy ranged from 6.7% to 7.6% and 96.1% to 101.4%. The mean assay variance of the six serum samples evaluated for incurred samples reanalysis for N-desmethyl-regorafenib-(pyridine)-N-oxide was 6.1%. The mean assay variance of the 13 CSF samples evaluated for incurred samples reanalysis for N-desmethyl-regorafenib-(pyridine)-N-oxide was 7.3%. No sample value beyond ±20% deviation from the mean was observed in human serum and CSF. Incurred sample reanalysis in human serum and CSF showed reliability of the assay for N-desmethyl-regorafenib-(pyridine)-N-oxide.

The precision and accuracy for the regorafenib-(pyridine)-N-oxide SQCs in human serum ranged from 2.9% to 8.6% and 99.5% to 102.5%. In human CSF, precision and accuracy ranged from 6.6% to 7.5% and 97.2% to 103.7%. The mean assay variance of the six serum samples evaluated for incurred samples reanalysis for regorafenib-(pyridine)-N-oxide was 8.5%. The mean assay variance of the 13 CSF samples evaluated for incurred samples reanalysis for regorafenib-(pyridine)-N-oxide was 5.3%. No sample value beyond ±20% deviation from the mean was observed in human serum and CSF. Incurred sample reanalysis in human serum and CSF showed reliability of the assay for regorafenib-(pyridine)-N-oxide.

## References

[1] Stupp R., Mason W.P., van den Bent M.J., Weller M., Fisher B., Taphoorn M.J., Belanger K., Brandes A.A., Marosi C., Bogdahn U. (2005). Radiotherapy plus concomitant and adjuvant temozolomide for glioblastoma. N. Engl. J. Med..

[2] Weller M., Cloughesy T., Perry J.R., Wick W. (2013). Standards of care for treatment of recurrent glioblastoma--are we there yet?. Neuro-Oncology.

[3] Ronellenfitsch M.W., Steinbach J.P., Wick W. (2010). Epidermal growth factor receptor and mammalian target of rapamycin as therapeutic targets in malignant glioma: Current clinical status and perspectives. Target. Oncol..

[4] Chinot O.L., Wick W., Mason W., Henriksson R., Saran F., Nishikawa R., Carpentier A.F., Hoang-Xuan K., Kavan P., Cernea D. (2014). Bevacizumab plus radiotherapy-temozolomide for newly diagnosed glioblastoma. N. Engl. J. Med..

[5] Batchelor T.T., Mulholland P., Neyns B., Nabors L.B., Campone M., Wick A., Mason W., Mikkelsen T., Phuphanich S., Ashby L.S. (2013). Phase III randomized trial comparing the efficacy of cediranib as monotherapy, and in combination with lomustine, versus lomustine alone in patients with recurrent glioblastoma. J. Clin. Oncol. Off. J. Am. Soc. Clin. Oncol..

[6] Hutterer M., Hattingen E., Palm C., Proescholdt M.A., Hau P. (2015). Current standards and new concepts in MRI and PET response assessment of antiangiogenic therapies in high-grade glioma patients. Neuro-Oncology.

[7] Wen P.Y., Macdonald D.R., Reardon D.A., Cloughesy T.F., Sorensen A.G., Galanis E., Degroot J., Wick W., Gilbert M.R., Lassman A.B. (2010). Updated response assessment criteria for high-grade gliomas: Response assessment in neuro-oncology working group. J. Clin. Oncol. Off. J. Am. Soc. Clin. Oncol..

[8] Wilhelm S.M., Dumas J., Adnane L., Lynch M., Carter C.A., Schutz G., Thierauch K.H., Zopf D. (2011). Regorafenib (BAY 73-4506): A new oral multikinase inhibitor of angiogenic, stromal and oncogenic receptor tyrosine kinases with potent preclinical antitumor activity. Int. J. Cancer.

[9] Grothey A., Van Cutsem E., Sobrero A., Siena S., Falcone A., Ychou M., Humblet Y., Bouche O., Mineur L., Barone C. (2013). Regorafenib monotherapy for previously treated metastatic colorectal cancer (CORRECT): An international, multicentre, randomised, placebo-controlled, phase 3 trial. Lancet.

[10] Demetri G.D., Reichardt P., Kang Y.K., Blay J.Y., Rutkowski P., Gelderblom H., Hohenberger P., Leahy M., von Mehren M., Joensuu H. (2013). Efficacy and safety of regorafenib for advanced gastrointestinal stromal tumours after failure of imatinib and sunitinib (GRID): An international, multicentre, randomised, placebo-controlled, phase 3 trial. Lancet.

[11] Bruix J., Qin S., Merle P., Granito A., Huang Y.H., Bodoky G., Pracht M., Yokosuka O., Rosmorduc O., Breder V. (2017). Regorafenib for patients with hepatocellular carcinoma who progressed on sorafenib treatment (RESORCE): A randomised, double-blind, placebo-controlled, phase 3 trial. Lancet.

[12] Hamed H.A., Tavallai S., Grant S., Poklepovic A., Dent P. (2015). Sorafenib/regorafenib and lapatinib interact to kill CNS tumor cells. J. Cell Physiol..

[13] Zhao P., Wang Y., Kang X., Wu A., Yin W., Tang Y., Wang J., Zhang M., Duan Y., Huang Y. (2018). Dual-targeting biomimetic delivery for anti-glioma activity via remodeling the tumor microenvironment and directing macrophage-mediated immunotherapy. Chem. Sci..

[14] Lombardi G., De Salvo G.L., Brandes A.A., Eoli M., Ruda R., Faedi M., Lolli I., Pace A., Daniele B., Pasqualetti F. (2019). Regorafenib compared with lomustine in patients with relapsed glioblastoma (REGOMA): A multicentre, open-label, randomised, controlled, phase 2 trial. Lancet Oncol..

[15] Stupp R. (2019). Drug development for glioma: Are we repeating the same mistakes?. Lancet Oncol..

[16] Kebir S., Rauschenbach L., Radbruch A., Lazaridis L., Schmidt T., Stoppek A.K., Pierscianek D., Stuschke M., Forsting M., Sure U. (2019). Regorafenib in patients with recurrent high-grade astrocytoma. J. Cancer Res. Clin. Oncol..

[17] Tzaridis T., Gepfner-Tuma I., Hirsch S., Skardelly M., Bender B., Paulsen F., Schaub C., Weller J., Schäfer N., Herrlinger U. (2019). Regorafenib in advanced high-grade glioma: A retrospective bicentric analysis. Neuro-Oncology.

[18] Capper D., Jones D.T.W., Sill M., Hovestadt V., Schrimpf D., Sturm D., Koelsche C., Sahm F., Chavez L., Reuss D.E. (2018). DNA methylation-based classification of central nervous system tumours. Nature.

[19] Ellingson B.M., Bendszus M., Boxerman J., Barboriak D., Erickson B.J., Smits M., Nelson S.J., Gerstner E., Alexander B., Goldmacher G. (2015). Consensus recommendations for a standardized Brain Tumor Imaging Protocol in clinical trials. Neuro-Oncology.

[20] Radbruch A., Lutz K., Wiestler B., Baumer P., Heiland S., Wick W., Bendszus M. (2012). Relevance of T2 signal changes in the assessment of progression of glioblastoma according to the Response Assessment in Neurooncology criteria. Neuro-Oncology.

[21] Rieger J., Bahr O., Muller K., Franz K., Steinbach J., Hattingen E. (2010). Bevacizumab-induced diffusion-restricted lesions in malignant glioma patients. J. Neuro-Oncol..

[22] Server A., Kulle B., Maehlen J., Josefsen R., Schellhorn T., Kumar T., Langberg C.W., Nakstad P.H. (2009). Quantitative apparent diffusion coefficients in the characterization of brain tumors and associated peritumoral edema. Acta Radiol.

[23] Patel P., Baradaran H., Delgado D., Askin G., Christos P., John Tsiouris A., Gupta A. (2017). MR perfusion-weighted imaging in the evaluation of high-grade gliomas after treatment: A systematic review and meta-analysis. Neuro-Oncology.

[24] Boxerman J.L., Schmainda K.M., Weisskoff R.M. (2006). Relative cerebral blood volume maps corrected for contrast agent extravasation significantly correlate with glioma tumor grade, whereas uncorrected maps do not. AJNR Am. J. Neuroradiol..

[25] Zopf D., Heinig R., Thierauch K.-H., Hirth-Dietrich C., Hafner F.-T., Christensen O., Lin T., Wilhelm S., Radtke M. (2010). Abstract 1666: Regorafenib (BAY 73-4506): Preclinical pharmacology and clinical identification and quantification of its major metabolites. Cancer Res..

[26] Zopf D., Fichtner I., Bhargava A., Steinke W., Thierauch K.H., Diefenbach K., Wilhelm S., Hafner F.T., Gerisch M. (2016). Pharmacologic activity and pharmacokinetics of metabolites of regorafenib in preclinical models. Cancer Med..

[27] Rieger J., Bahr O., Ronellenfitsch M.W., Steinbach J., Hattingen E. (2010). Bevacizumab-induced diffusion restriction in patients with glioma: Tumor progression or surrogate marker of hypoxia?. J. Clin. Oncol. Off. J. Am. Soc. Clin. Oncol..

[28] Gerisch M., Hafner F.T., Lang D., Radtke M., Diefenbach K., Cleton A., Lettieri J. (2018). Mass balance, metabolic disposition, and pharmacokinetics of a single oral dose of regorafenib in healthy human subjects. Cancer Chemother. Pharm..

[29] Collins J.M., Dedrick R.L. (1983). Distributed model for drug delivery to CSF and brain tissue. Am. J. Physiol..

[30] De Lange E.C., Danhof M. (2002). Considerations in the use of cerebrospinal fluid pharmacokinetics to predict brain target concentrations in the clinical setting: Implications of the barriers between blood and brain. Clin. Pharmacokinet..

[31] Pope W.B., Xia Q., Paton V.E., Das A., Hambleton J., Kim H.J., Huo J., Brown M.S., Goldin J., Cloughesy T. (2011). Patterns of progression in patients with recurrent glioblastoma treated with bevacizumab. Neurology.

[32] Nowosielski M., Ellingson B.M., Chinot O.L., Garcia J., Revil C., Radbruch A., Nishikawa R., Mason W.P., Henriksson R., Saran F. (2018). Radiologic progression of glioblastoma under therapy-an exploratory analysis of AVAglio. Neuro-Oncology.

[33] Ganau M., Paris M., Syrmos N., Ganau L., Ligarotti G.K.I., Moghaddamjou A., Prisco L., Ambu R., Chibbaro S. (2018). How Nanotechnology and Biomedical Engineering Are Supporting the Identification of Predictive Biomarkers in Neuro-Oncology. Medicines (Basel).

[34] Ganau L., Prisco L., Ligarotti G.K.I., Ambu R., Ganau M. (2018). Understanding the Pathological Basis of Neurological Diseases Through Diagnostic Platforms Based on Innovations in Biomedical Engineering: New Concepts and Theranostics Perspectives. Medicines (Basel).

[35] Jost G., Pietsch H., Grenacher L. (2013). Dynamic contrast-enhanced computed tomography to assess antitumor treatment effects: Comparison of two contrast agents with different pharmacokinetics. Invest Radiol..

[36] Mong S., Ellingson B.M., Nghiemphu P.L., Kim H.J., Mirsadraei L., Lai A., Yong W., Zaw T.M., Cloughesy T.F., Pope W.B. (2012). Persistent diffusion-restricted lesions in bevacizumab-treated malignant gliomas are associated with improved survival compared with matched controls. AJNR Am. J. Neuroradiol..

[37] Cachia D., Elshafeey N.A., Kamiya-Matsuoka C., Hatami M., Alfaro-Munoz K.D., Mandel J.J., Colen R., DeGroot J.F. (2017). Radiographic patterns of progression with associated outcomes after bevacizumab therapy in glioblastoma patients. J. Neuro-Oncol..

[38] Gupta A., Young R.J., Karimi S., Sood S., Zhang Z., Mo Q., Gutin P.H., Holodny A.I., Lassman A.B. (2011). Isolated diffusion restriction precedes the development of enhancing tumor in a subset of patients with glioblastoma. AJNR Am. J. Neuroradiol..

[39] Bahr O., Harter P.N., Weise L.M., You S.J., Mittelbronn M., Ronellenfitsch M.W., Rieger J., Steinbach J.P., Hattingen E. (2014). Sustained focal antitumor activity of bevacizumab in recurrent glioblastoma. Neurology.

[40] Ellingson B.M., Cloughesy T.F., Lai A., Nghiemphu P.L., Lalezari S., Zaw T., Motevalibashinaeini K., Mischel P.S., Pope W.B. (2012). Quantification of edema reduction using differential quantitative T2 (DQT2) relaxometry mapping in recurrent glioblastoma treated with bevacizumab. J. Neuro-Oncol..

