# Supplementary material for: Regorafenib CSF Penetration, Efficacy, and MRI Patterns in Recurrent Malignant Glioma Patients
Source: J Clin Med. 2019 Nov 21;8(12):2031. doi: 10.3390/jcm8122031 (PMC6947028; doi:10.3390/jcm8122031)
Supplement: Supplementary file 1 [file jcm-08-02031-s001.pdf]

Supplementary Information

Regorafenib CSF penetration, efficacy and MRI patterns in recurrent malignant glioma patients

Pia S. Zeiner et al.

Supplementary Table 1

Patient No.	Days REG	RANO	Growth Pattern	CE Tumor	NE Tumor	Edema	$rCBV_{max}$ follow-up *(baseline)*	ADC_{ratio} follow-up *(baseline)*	CT	WHO°	IDH*	MGMT
1	12‡	PD	T2-dom.	(-)	(+)	(+)*	8.1 *(N/A)*	0.42 *(1.00)*	y	IV	wt	met
2	40	PD	T2-dom.	(-)	(+)	(+)*	N/A *(N/A)*	N/A *(0.98)*	n	III	mut	N/A
4	57	PD	T2-dom.	(-)	(+)	(+)*	N/A *(N/A)*	0,54 *(0.89)*	y	IV	wt	met
7	62	PD	T2-dom.	(-)	(+)	(-)	N/A *(N/A)*	0.55 *(1.14)*	y	IV	mut	met
10	77‡	PD	T2-dom.	(-)	(+)	(+)	1.0 *(0.7)*	0.49 *(N/A)*	n	IV	wt	met
19	63	PD	T2-dom.	(-)	(+)	(-)	N/A *(N/A)*	0.61 *(1.04)*	n	III	wt	N/A
15	49‡	PD	T2-dom.	(-)	(+)	(-)	3.3 *(N/A)*	0.59 *(1.40)*	n	IV	wt	non
20	59	PD	T2-dom.	(-)	(+)	(+)*	4.0 *(1.0)*	0.42 *(1.40)*	y	IV	wt	non
8	23‡	SD	T2-dom.	(-)	(+)	(+)	1.1 *(3.1)*	0.73 *(1.41)*	y	IV	wt	met
9	12‡	PD	T2-dom.	(-)	(+)	(+)	3.2 *(4.4)*	0.75 *(1.25)*	y	III	mut§	met
21	49	PD	T2-dom.	(-)	(+)	(-)	3.0 (5.1)	0.41 (0.95)	y	IV	wt	non
3	28	PD	classic	(+)	(+)	(+)	3.4 *(N/A)*	0.96 *(1.00)*	y	III	mut	N/A
5	44	PD	classic	(+)	(+)	(-)	6.7 *(4.2)*	0.91 *(1.04)*	y	IV	wt	met
11	36	PD	classic	(+)	(+)	(-)*	2.5 *(2.6)*	0.58 *(0.87)*	n	IV	wt	non
14	52	PD	classic	(+)	(+)	(+)	2.5 *(4.6)*	0.57 *(0.66)*	n	IV	wt	N/A
17	56	PD	classic	(+)	(+)	(+)	N/A *(N/A)*	0.65 *(1.00)*	y	IV	wt	non
18	20	PD	classic	(+)	(+)	(+)	2.5 *(N/A)*	0.83 *(0.99)*	y	IV	mut	met
13	52	PD	classic	(+)	(+)	(-)	4.6 *(N/A)*	0.87 *(0.95)*	y	IV	wt	non
16	64‡	SD	Not classified	(0)	(0)	(0)	*(N/A)*	1.45 (1.75)	n	IV	N/A	N/A

*** determined by IDH1 R132H immunoreactivity**

Supplementary Table 1. Patient and imaging characteristics for first magnetic resonance imaging (MRI) follow-up during regorafenib (REG) treatment.

Patient No. = Patient number, Days REG = Days from start of regorafenib treatment to first follow-up MRI, ‡ = At least one more follow-up MRI available, RANO = Diagnosis based on the revised assessment in neuro-oncology criteria, PD = Progressive disease, SD = Stable disease, T2-dom. = T2 dominant, CE Tumor = Contrast-enhancing tumor, NE Tumor = Non-enhancing tumor, (-) = Overall decrease, (+) = Overall increase, * = Simultaneous de- and increase of edema in different lesions with the sign in brackets indicating the overall development, $rCBV_{max}$ = Relative maximum cerebral blood

volume in the tumor, ADC_{ratio} = Ratio of minimum apparent diffusion coefficient (ADC) in the tumor and ADC in the normal appearing, contralateral tissue, CT = CT scan available, y = yes, n = no, WHO° = WHO grade, IDH = isocitrate dehydrogenase, mut = mutant, wt = wildtype, § = additional 1p/19q co-deletion, MGMT = O6-methylguanine-DNA methyltransferase promoter status, met = methylated, non = non-methylated, N/A = Not available or not evaluable.

Supplementary Figures

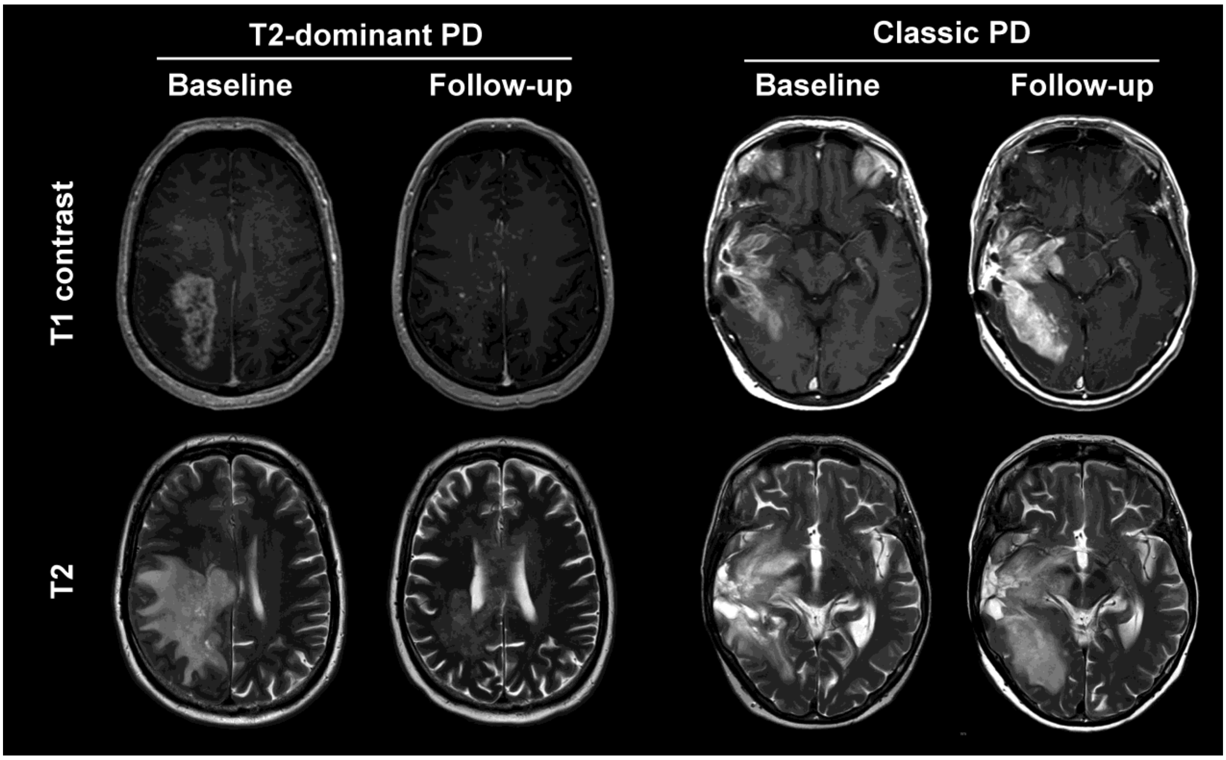

Supplementary Figure 1. Patterns of malignant glioma progression.

T2-dominant (patient 7) and classic (patient 13) progressive disease patterns with corresponding T1 contrast-enhanced (T1 contrast) and T2-weighted (T2) MR images.

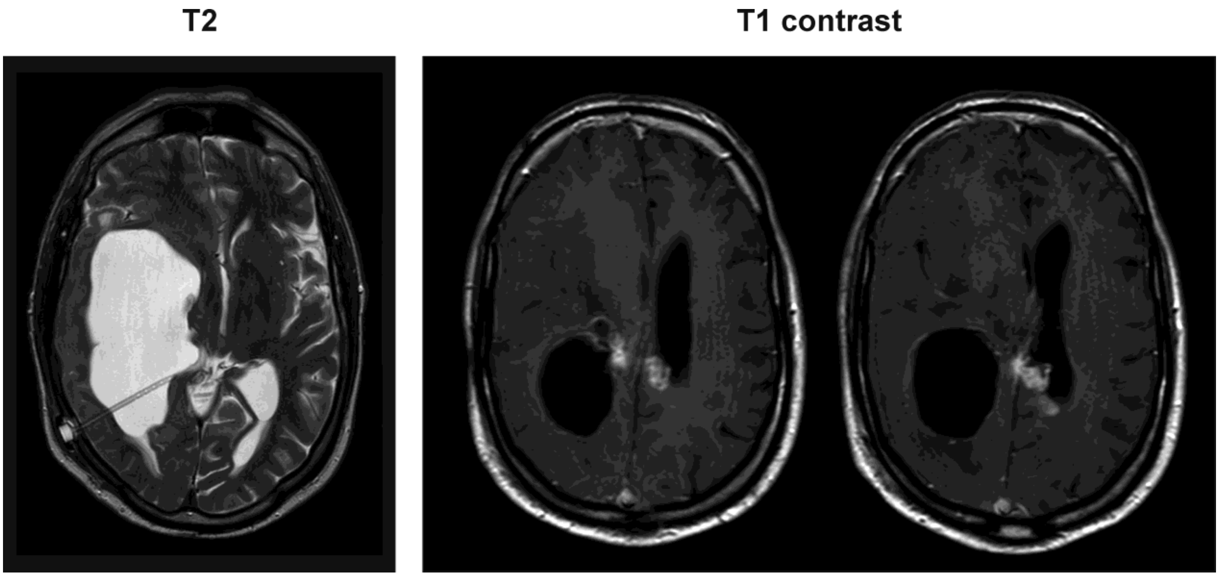

Supplementary Figure 2. Rickham reservoir implantation in a patient with occlusion hydrocephalus due to a glioblastoma.

Magnetic resonance imaging (MRI) T2 sequence of patient 5 with Rickham reservoir (left panel) and T1 contrast-enhanced image of a different slice of patient 5 displaying contrast-enhancing tumor tissue prior to initiation of regorafenib therapy (right panel).

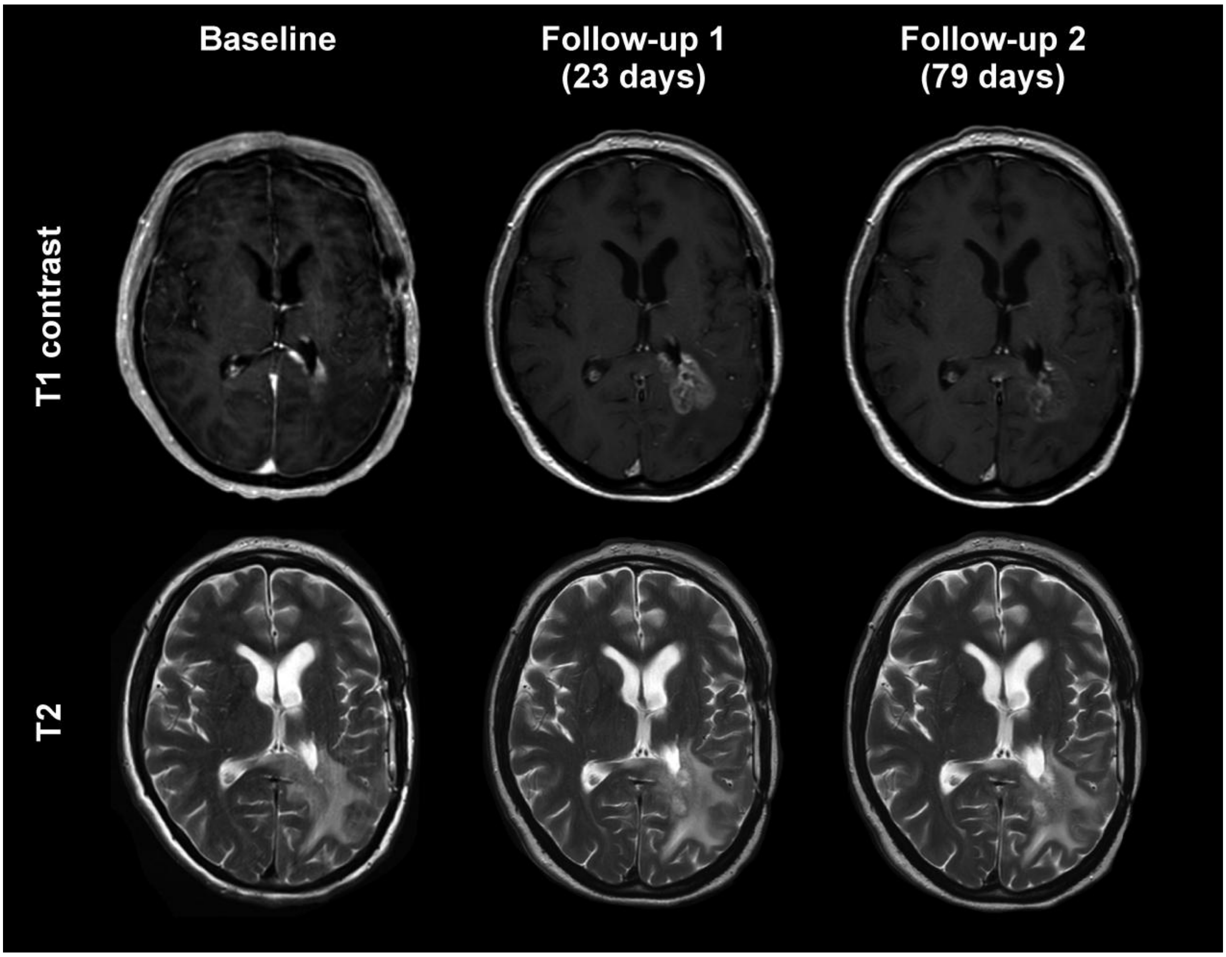

Supplementary Figure 3. Magnetic resonance imaging dynamics of patient 8.

Corresponding T1 contrast-enhanced (T1 contrast) and T2-weighted (T2) MRI for baseline and first follow-up (follow-up 1, 23 days after the start of regorafenib (REG) treatment) and second follow-up (follow-up 2, 79 days after the start of REG treatment). Notice how the contrast enhancement is decreasing and the tumor growth almost comes to a hold when comparing follow-up 1 to follow-up 2.

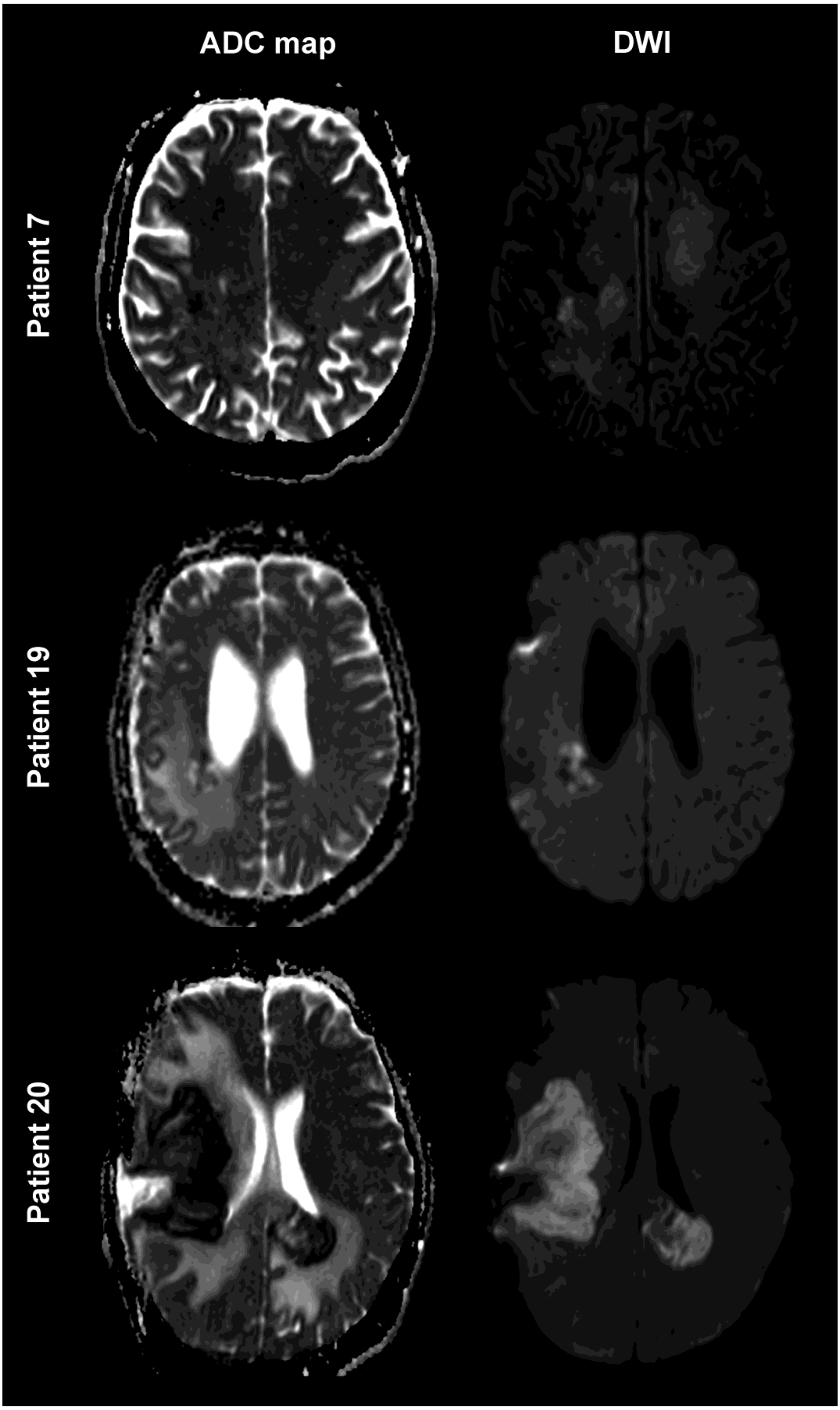

Supplementary Figure 4. Stroke-like lesions in regorafenib-treated patients

Exemplary magnetic resonance imaging (MRI) of 3 patients with apparent diffusion coefficient maps (ADC map) on the left and corresponding b 1000 diffusion weighted images (DWI) on the right.
